# Subtypes of complex regional pain syndrome—a systematic review of the literature

**DOI:** 10.1097/PR9.0000000000001111

**Published:** 2023-11-15

**Authors:** Lone Knudsen, Lana Santoro, Stephen Bruehl, Norman Harden, Florian Brunner

**Affiliations:** a National Rehabilitation Center for Neuromuscular Diseases, Aarhus, Denmark; b Wheelock College of Education and Human Development, Boston University, Boston, MA, USA; c Department of Anesthesiology, Vanderbilt University Medical Center, Nashville, TN, USA; d Northwestern University, Chicago, IL, USA; e Department of Physical Medicine and Rheumatology, Balgrist University Hospital, Zurich, Switzerland

**Keywords:** Complex regional pain syndrome, Subtypes, Subgroups

## Abstract

To systematically identify and summarize possible subtypes of complex regional pain syndrome (CRPS), we searched MEDLINE, Embase, Cochrane, Scopus, and Web of Science for original studies reporting or investigating at least one subtype within a group of patients with CRPS. The search retrieved 4239 potentially relevant references. Twenty-five studies met our inclusion criteria and were included in the analysis. Complex regional pain syndrome phenotypes were investigated based on the following variables: clinical presentation/sensory disturbances, dystonia, skin temperature, disease duration, onset type, CRPS outcome, and neuropsychological test performance. Support was found for the following CRPS subtypes: CRPS type I, CRPS type II, acute CRPS, chronic CRPS, centralized CRPS, cold CRPS, warm CRPS, inflammatory CRPS, dystonic CRPS, nondystonic CRPS, familial CRPS, and nonfamilial CRPS. It is unclear whether these are distinct or overlapping subtypes. The results of this comprehensive review can facilitate the formulation of well-defined CRPS subtypes based on presumed underlying mechanisms. Our findings provide a foundation for establishing and defining clinically meaningful CRPS subtypes, with the ultimate goal of developing targeted and enhanced treatments for CRPS.

## Abstract 2

This comprehensive review of 25 studies explores variables to identify and define subtypes of complex regional pain syndrome, aiding the development of targeted treatments.

## 1. Introduction

Complex regional pain syndrome (CRPS) is a primary pain condition that is characterized by disproportional pain after an initiating noxious event such as trauma or surgery.^28^ Clinical manifestations are heterogenous and include sensory (allodynia, hyperpathia), vasomotor and sudomotor (temperature asymmetry, skin color changes, edema, sweating asymmetry), motor (decrease of range of motion, weakness, tremor, dystonia), and trophic changes of the hair, nails, and skin in the affected limb.^28^ Although favorable prognosis has been described in some,^33^ many patients develop chronic CRPS with persistent pain and functional disability.^3^

From its inception as a diagnostic term in 1994, CRPS was intended to serve as an umbrella diagnosis that encompassed a variety of painful disorders.^40^ In the past, several attempts have been made to classify this heterogenous condition into meaningful clinical subtypes. For example, the original 1994 diagnostic nomenclature specified 2 subtypes reflecting the previous distinction between older diagnoses of reflex sympathetic dystrophy and causalgia: CRPS type I (without major peripheral nerve damage) and CRPS type II (with major peripheral nerve damage), respectively.^40^ However, despite increasing research in the field of CRPS, current treatment regimens remain inadequate, and it is unclear whether the CRPS type I vs CRPS type II subtype distinction meaningfully affects treatment outcomes.^8^

It is possible that to date, the lack of significant efficacy in large pharmaceutical trials may arise because of the existence of multiple CRPS subtypes in trial samples selected based on current broad diagnostic criteria (eg, the new IASP criteria, formerly known as the Budapest criteria) that dilute beneficial treatment effects for a responsive patient subtype when grouped together. Different disease mechanisms may underlie different CRPS clinical phenotypes (ie, subtypes) and therefore may require different types of treatments.^6^ To develop more effective CRPS treatments, it is critical to identify relevant CRPS subtypes (ideally reflecting CRPS mechanisms) that specific treatments with known mechanisms of action can target (ie, precision medicine). Several subtypes have been proposed in the literature, but no systematic summary of the evidence is available. Hence, the aim of this review was to systematically identify and summarize the evidence for subtypes in CRPS.

## 2. Methods

This systematic review was conducted in accordance with the recommendations by the Preferred Reporting Items for Systematic Reviews and Meta-Analyses (PRISMA statement),^45^ and the study was registered in the Open Science Framework (https://osf.io).

### 2.1. Search strategy

We performed a comprehensive electronic search in MEDLINE (OvidSP), Embase (Elsevier), Cochrane, Scopus, and Web of Science from inception to July 2022 to identify relevant references. The terms for the search strategies were identified through discussion between an information specialist and the review team, by scanning the background literature and by browsing the MEDLINE Thesaurus (MeSH). The combinations of the following search keywords and MeSH terms were used in title, abstract and keywords: “reflex sympathetic dystrophy,” “complex regional pain syndrome,” ‘CRPS,” “algodystrophy,” “Sudeck” AND “subgroup,” “subtype,” “subset,” “phenotype,” “cluster.” The search was performed by a professional information specialist. To ensure the completeness of the literature search, 2 reviewers who were experienced clinicians and researchers in the field of CRPS screened bibliographies of all included studies and retrieved articles in an additional hand search. All potentially eligible references were included in the full-text review (inclusion criteria applied).

### 2.2. Study selection and main outcome variables

The eligibility criteria for inclusion in the current systematic review were (1) human studies, (2) established diagnosis of CRPS by applying disease-specific diagnostic criteria, and (3) reporting of at least one subtype (or subgroup, subset, phenotype or cluster) within a CRPS sample. We applied no language restriction. Opinion papers, letters, conference abstracts and review articles were excluded.

### 2.3. Risk of bias assessment

The quality of all studies was assessed using the MINORS criteria (methodological items for nonrandomized studies).^48^ These criteria assess 8 critical aspects of study design for noncomparative clinical studies and an additional 4 aspects of study design for comparative clinical studies. Each item is given a score of zero if information is not reported, one if information is reported but inadequate, and 2 if information is reported and adequate. The sum score results in a maximum score of 16 for noncomparative studies and 24 for comparative studies. Each score was then converted into a percentage to harmonize the scoring system. Depending on the score achieved, studies were qualified as either having low-risk of bias (≥75%), moderate-risk of bias (50% ≤ score <75%), or high-risk of bias (<50%).

### 2.4. Analysis

Citations from the initial search results of each database were exported to EndNote (version X9.2, Clarivate Analytics, Philadelphia, PA), and duplicates were removed. The titles and abstracts were screened and reviewed by 2 authors (L.K. and F.B.). Then, full texts of potential studies were retrieved and independently reviewed in detail for inclusion based on the predetermined criteria. Discrepancies between the 2 authors were resolved by discussion, and a third author (S.B.) was consulted if consensus could not be reached.

One author (F.B.) extracted the data from the included studies into a piloted standardized data collection form, and another author (L.K.) cross-checked the extracted data. The following data were extracted: author, year, study design, diagnostic criteria, number of participants with CRPS and their age, sex, site of CRPS, and disease duration. Subtypes were investigated or identified, and the main findings were also extracted.

## 3. Results

### 3.1. The flow of study selection

**Figure 1.**
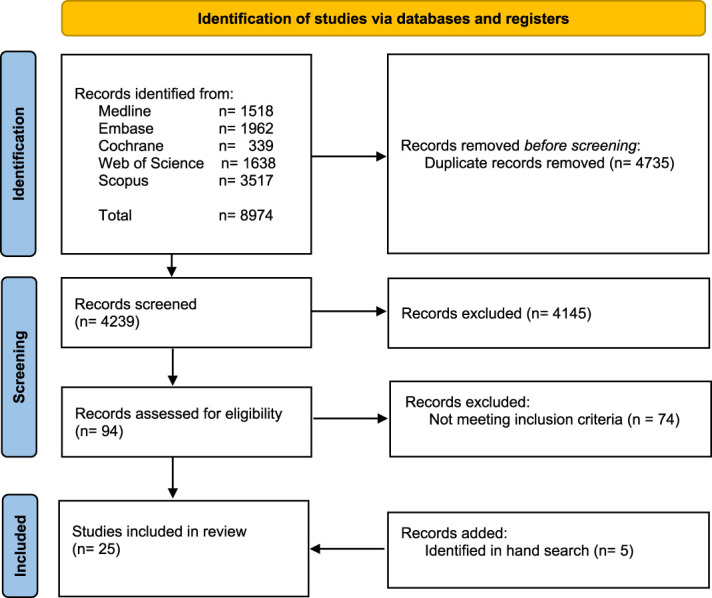
Study selection according to the Preferred Reporting Items for Systematic Reviews and Meta-Analysis (PRISMA) flowchart.

The search retrieved 8974 total records. With duplicates removed before screening, there were 4239 potentially relevant references. After screening titles and abstracts, the full text of 94 abstracts was reviewed. Of these, 74 references were excluded because they did not meet the inclusion criteria, leaving 20 included studies. Five additional studies were added based on the hand search.^4,26,30,51,52^ In total, 25 studies were included in the final analysis. Figure 1 provides the PRISMA flow diagram.

### 3.2. Characteristics of the included studies and the patients

The characteristics of the studies and the patient samples for each are summarized in Table 1. The studies were published between 2002 and 2022. In the majority of studies, the diagnosis was based on the current International Association for the Study of Pain (Budapest) criteria (N = 16).^1,4,9,13,18–23,25,30,38,39,55,57^ Most studies employed a cross-sectional (N = 16)^4,11,14,15,20,21,23,25,26,30,39,51–55^ or cohort (N = 6)^1,8,9,13,18,38^ design. Some of the studies limited the inclusion to patients with CRPS type I (N = 8),^8,11,30,51–55^ and some studies did not report whether patients had CRPS type I or CRPS type II (N = 6).^14,15,19,25,38,57^ The majority of studies included patients with upper and lower extremity CRPS (N = 15).^4,8,9,11,13–15,18,20,21,23,25,39,55,57^ A total of 4908 patients (mean of 207 per study, range 37–1037) with a mean age of 46.9 years (SD 5.1) were included in the analysis. All studies predominantly included female participants (mean 68.5%) consistent with the sex difference previously noted in CRPS prevalence.^12,46^ The mean disease duration across studies ranged from 4 months^4,25^ to 9.3 years^53^ (mean, 2.9 years).

**Table 1:** Characteristics of the included studies.

Author, y (reference)	Study design	Diagnostic criteria	No. of participants with CRPS N, n	Age (y)	Female (%)	Site	Disease duration	Subgroups	Description
Alexander et al., 2012^1^	Cluster analysis	IASP/Budapest	N = 148 CRPS type I: 100 CRPS-type II: 48	44.5 (range 20–68)	80	NR	8.8 y (range 0.7–36)	Two subgroups: (1) Subgroup similar to healthy controls (2) Subgroup with elevated levels of IL-1β, IL-4, IL-7, TNF-α, sIL-1RI, sIL-2Rα, sTNF-RI, sTNF-RII, IL-1Ra, and MCP-1	Evaluation of plasma levels of cytokines, chemokines, and their soluble receptors in CRPS patients vs gender-matched and age matched healthy controls Significant changes were found in CRPS patients compared with healthy controls Two clusters were identified in CRPS subjects TNF-α was the most important category for cluster separation When present, increased plasma levels of TNF-alpha correlated with disease duration and severity
Birklein et al., 2014^4^	Cross sectional	IASP/Budapest	N = 55 CRPS type I: 44 CRPS type II: 11	49.8 (SEM 1.8, range 20–72)	67	UE, LE	16 wk (range 3–920)	Two subgroups: (1) Acute CRPS (<3 mo) (2) Chronic CRPS (>3 mo)	Detection of keratinocyte and mast cell proliferation in skin biopsies from the CRPS limb and the contralateral limb and identification of the cellular source of the upregulated TNF-alpha, IL-6, and tryptase Epidermal thickness and keratinocyte expression were increased in acute CRPS skin and decreased in chronic CRPS skin. Increases in TNF-alpha and IL-6 immunostaining were more frequent in acute CRPS skin than in chronic CRPS skin. Tryptase-positive dermal mast cell numbers were also increased in acute CRPS skin but similar to contralateral skin in chronic CRPS skin. Numbers of tryptase-labelled mast cells correlated positively with temperature asymmetry (warm CRPS limb)
Bruehl et al., 2002^8^	Cross sectional, cohort, cluster analysis	IASP/Orlando	N = 113 CRPS type I: 113 CRPS type II: 0	41.3 (SD 9.9)	62.5	UE, LE	26.9 mo (SD 28.8)	Three subgroups: (1) Vasomotor subgroup (2) Neuropathic/sensory abnormalities subgroup (3) Florid CRPS subgroup	Cluster analysis based on signs and symptoms of the diagnostic criteria resulted in the 3 subgroups Identified subgroups did not differ significantly regarding pain duration. Rate of abnormalities on EMG/nerve conduction studies was most common in the neuropathic/sensory abnormalities subgroup
Bruehl et al., 2016^9^	Prospective, between-subject, and within-subject design, 3 mo follow-up, cluster analysis	IASP/Budapest	N = 152 CRPS type I: 129 CRPS type II2: 23	46.4 (SD 13.4)	74.2	UE, LE	4.7 mo (warm CRPS) 20 mo (cold CRPS)	Two subgroups: (1) Warm CRPS (2) Cold CRPS	Cluster analysis based on signs and symptoms of inflammation resulted in the 2 subgroups Subgroups did not differ significantly regarding pain intensity Pain duration was shorter in warm CRPS (4.7 mo) than in cold CRPS (20 mo). Inflammatory score was elevated in warm CRPS and decreased over the 3-mo follow-up in warm CRPS but did not change in cold CRPS
De Boer et al., 2011^11^	Cross sectional	IASP/Orlando	N = 681 CRPS type I: 681 CRPS type II: 0	47.2 (SD 13.8)	82.8	UE, LE	14.4 (IQR 3.6–73.2)	Four subgroups based on disease duration: (1) <2 mo (2) 2–6 mo (3) 6–12 mo (4) >12 mo	Description and assessments of signs and symptoms in relation to disease duration Sensory signs (allodynia, hyperalgesia) and motor signs (except limited range of motion) occurred more in patients with a longer disease duration (>6 mo) than in patients with a shorter disease duration Vasomotor and sudomotor signs were less frequent in patients with longer duration (>6 mo) Changes in nail growth (but not skin or hair growth) were more frequent in patients with longer disease duration
De Mos et al., 2009^13^	Cluster analysis	IASP/Budapest	N = 102 CRPS type I: 99 CRPS type II: 3	51 (12–86)	79	UE, LE	5.8 y (range: 2.1–10.8)	Three subgroups: (1) Best-outcome subgroup (2) Moderate-outcome subgroup (3) Poor-outcome subgroup	Retrospective analysis of electronic patient records with CRPS patients and reference patients with an identical past injury but without CRPS. Patients with poor outcome more often had cold upper extremity CRPS with initiating event other than fracture. Disease duration did not differ between the groups More patients in the poor-outcome group (based on the number of signs and symptoms) reported ongoing disease progression, ongoing treatment, and poorer work status
De Rooij et al., 2009^14^	Cross sectional	IASP/Orlando	N = 271 CRPS type I: NR CRPS type II: NR	36.7 (SD 14.5)	83	UE, LE	NR	Two subgroups: (1) Familial CRPS subgroup (2) Sporadic CRPS subgroup	Evaluation of familial occurrence in CRPS patients Familial CRPS patients had younger age at onset, multiple affected extremities, dystonia
De Rooij et al., 2010^15^	Cross sectional	IASP/Orlando	N = 537 CRPS type I: NR CRPS type II: NR	42.5 (SD 15.6)	78	UE, LE	1.1 y (IQR 0.3–2.8)	Two subgroups: (1) Spontaneous onset of CRPS (2) Trauma-induced onset of CRPS	Comparison of phenotypic characteristics of patients with spontaneous and trauma-induced CRPS Patients with spontaneous onset were younger at onset and had a longer disease duration. Clinical presentation was similar in the 2 groups
Dimova et al., 2020^18^	Cluster analysis	IASP/Budapest (research criteria)	N = 1037 CRPS type I: 890 CRPS type II: 147	51.7 (SD 13.1)	72.5	UE, LE	9.4 mo (SD 15.8)	Three subgroups: (1) Peripheral inflammation phenotype (2) Central phenotype (3) Mixed phenotype	Cluster analysis of CRPS signs resulted in the 3 subgroups The phenotypes may reflect major pathophysiologic mechanisms of peripheral inflammation and central changes The 3 subgroups did not differ in relation to age, CRPS duration, sex, or CRPS type I or II
Dirckx et al., 2015^19^	Retrospective	IASP/Budapest	N = 48 CRPS type I: NR CRPS type II: NR	47.8 (SD 10.0)	66.7	NR	6 mo (IQR 12.75)	Three subgroups: (1) Warm CRPS (ΔT ≥ 0.60°C in CRPS limb vs healthy limb) (2) Cold CRPS (ΔT ≥ 0.60°C lower in CRPS limb vs healthy limb) (3) Intermediate CRPS (neither cold nor warm)	Assessment of signs of inflammation in warm, cold, and intermediate CRPS No difference in TNF-alpha and IL-6 in artificial skin blisters on the affected and nonaffected sides Longer CRPS duration and lower age in cold CRPS than in warm CRPS Cold CRPS and intermediate CRPS more often had signs of allodynia
Drummond et al., 2014^20^	Cross sectional	IASP/Budapest	N = 37 CRPS type I: 29 CRPS type II: 8	47.2 (SD 11.1)	78.3	UE, LE	52.7 mo (SD 58.8)	Chronic CRPS (pain duration = 66 ± 14 mo)	To determine whether a central disturbance in somatosensory processing contributes to limb pain in CRPS. Increase of pain in the CRPS limb when ipsilateral forehead was cooled in a subgroup with a longer pain duration and more pain in the CRPS limb
Drummond et al., 2018^21^	Cross sectional	IASP/Budapest (research criteria)	N = 104 CRPS type I: 71 CRPS type II: 33	46.8 (SD 11.2)	70.2	UE, LE	49.0 mo (SD 27.0)	Two subgroup analyses (A, B) (A) 2 subgroups: (1) CRPS type I (2) CRPS type II and (B) 3 subgroups: (1) Acute CRPS (<12 mo) (2) Intermediate CRPS (13–36 mo) (3) Chronic CRPS (>36 mo)	Assessing hemisensory disturbances in CRPS patients Increase in sharpness ratings to repeated pinprick was greater in the ipsilateral than in contralateral forehead in chronic but not in intermediate and acute CRPS Patients with chronic CRPS were more sensitive to thermal stimuli on both sides of their body than patients with acute or intermediate CRPS Cold-pain ratings were greater on the ipsilateral than on the contralateral side of the forehead in patients with chronic CRPS but not in patients with acute or intermediate CRPS Tactile detection threshold to graded von Frey hairs was elevated in the CRPS 2 limb compared with contralateral limb but was similar in the affected and contralateral limb of patients with CRPS type I Also, sensitivity to pinprick was lower in the CRPS limb of CRPS type II than in the CRPS type I patients Brush-evoked allodynia was more common in the CRPS type II limb than in the CRPS type I limb Patients with chronic CRPS type I more often had pressure-pain sensitivity and allodynia in the uninjured limb on the symptomatic side of the body than in patients with chronic CRPS type II
Drummond et al., 2018^22^	Double-blind crossover trial	IASP/Budapest (research criteria)	N = 90 CRPS type I: 59 CRPS type II: 31	47.1 (SD 11.3)	70	UE	52 mo (SD 70 mo)	Three subgroup analyses (A, B, C) (A) 2 subgroups (1) Warm CRPS (≥1.0°C higher in CRPS limb vs healthy limb) (2) Cold CRPS (≥1.0°C lower in CRPS limb vs healthy limb) and (B) 3 subgroups: (1) Acute CRPS (<12 mo) (2) Intermediate CRPS (13–36 mo) (3) Chronic CRPS (>36 mo) and (C) 2 subgroups: (1) CRPS type I (2) CRPS type II	To evaluate whether upregulated cutaneous expression of α1-adrenoceptors is a source of pain in CRPS Skin biopsies of the CRPS limb and the contralateral limb were assessed for adrenoceptors Evoked pain and responses to pinprick was investigated to intradermal injections of α1-adrenoceptor agonist phenylephrine or α2-AR agonist clonidine in the CRPS limb and the contralateral limb No differences were seen between thermal groups α1-AR immunoreactivity was greater in nerve bundles bilaterally in CRPS patients than in controls and was greater in dermal nerves in the CRPS limb vs contralateral limb irrespective of CRPS duration. However, α1-AR immunoreactivity was greater in nerve bundles of the CRPS limb in patients with acute than intermediate or chronic CRPS but similar across duration in the contralateral limb There was greater α1-AR immunoreactivity in nerve bundles of the CRPS 2 limb than in those of the CRPS type I limb. Greater α1-AR immunoreactivity was also seen in blood vessels in both limbs of CRPS type II than in the limbs of CRPS type I patients A difference in α1-AR immunoreactivity in the epidermis was seen with disease duration in CRPS type I and type II. For CRPS type II, it was greater bilaterally in acute and intermediate vs chronic, but the opposite was seen in CRPS type I A group of phenylephrine responders emerged across subgroups who became more sensitive to pinprick and had greater α1-AR in dermal nerve bundles
Eberle et al., 2009^23^	Cross sectional	IASP/Budapest	N = 50 CRPS type I: 46 CRPS type II: 4	46.0 (SD 9)	92	UE, LE	21.5 wk (SD 3.0)	Two subgroups: (1) Warm CRPS (mean ΔT = +1.0° in CRPS limb vs healthy limb) (2) Cold CRPS (mean ΔT = −1.0°C in CRPS limb vs healthy limb)	Investigation of clinical differences in warm and cold CRPS Cold CRPS patients more often reported a history of serious life events, chronic pain disorders, dystonia, cold-induced pain, and sensory loss to QST Warm CRPS was characterized by mechanical hyperalgesia
Escolano et al., 2021^25^	Cross sectional	IASP/Budapest	N = 38 CRPS type I: NR CRPS type II: NR	48.0	62.3	UE, LE	16.9 wk	MMP-2 MMP-9	Comparison of MMP-2 and MMP-9 concentrations in skin and serum of patients with CRPS, other pain diseases, and healthy subjects Low ipsilateral MMP-2 was associated with trophic changes Contralateral MMP-2 was associated inversely with CRPS severity Higher MMP-9 was associated with higher CRPS severity
Gierthmühlen et al., 2012^26^	Cross sectional	IASP/Orlando and IASP/Budapest	N = 344 CRPS type I: 298 CRPS type II: 46	52.7 (SD 12.7)	78.8	UE	23.2 (SD 34.4)	Two subgroups: (1) CRPS type I 2) CRPS type II	To investigate somatosensory signs in CRPS type I, CRPS type II, and peripheral nerve injury using QST CRPS types I and II had similar somatosensory profiles in terms of hyperalgesia, allodynia, and thermal detection thresholds, but a loss of mechanical detection was more frequent in CRPS type II. Pressure hyperalgesia was most frequent in both CRPS types I and II compared with peripheral nerve injury. Hyperalgesia/allodynia without the loss of detection (only gain) was more common in CRPS type I than in CRPS type II. Correlations with symptom duration were mild. Small fiber deficits were less frequent than large fiber deficits
Huge et al., 2008^30^	Cross sectional	IASP/Budapest (research criteria)	N = 65 CRPS-type I: 65 CRPS-type II: 0	59.1 (SD 12.9)	88.5	UE	22.4 (SD 20.4)	Two subgroups: (1) Acute CRPS (≤12 mo) (2) Chronic CRPS (>12 mo)	Investigation of thermal QST in acute and chronic CRPS patients vs a group of gender-matched and age-matched healthy controls Patients with acute CRPS experienced warm and cold hyperalgesia and warm and cold hypoesthesia in the CRPS limb. Thermal hyperalgesia was lower in chronic CRPS, and warm and cold hypoesthesia was worse in chronic than in acute CRPS. Only patients with acute CRPS had paradoxical heat sensations. All QST changes were somewhat present in the contralateral limb Patients with chronic CRPS had a cold CRPS limb and patients with acute CRPS a warm CRPS limb
Libon et al., 2010^38^	Cluster analysis	IASP/Budapest (research criteria)	N = 137 CRPS type I: NR CRPS type II: NR	43.8 (SD 11.9)	77.9	NR	NR	Three subgroups based on cognitive function: (1) Normal (2) Dysexecutive (3) Global dysfunction	To elucidate the existence of neuropsychological subtypes using tests that assess executive control, naming/lexical retrieval, and declarative memory. Limb pain and depression were also assessed Cognitive function subgroups did not differ in level of pain, CRPS duration, medication use, or number of limbs with CRPS. Neuropsychological variables did not covary with depression
Lunden et al., 2022^39^	Cross sectional	IASP/Orlando and IASP/Budapest	N = 61 CRPS type I: 43 CRPS type II: 18	38.7 (spread not reported)	45.9	UE, LE	5 y (spread not reported)	Three subgroups: (1) Thermal allodynia (2) Thermal hyposensitivity (3) Thermal allodynia und hyposensitivity	Investigation of whether the 3 QST subgroups differ with respect to limb pain characteristics Paroxysmal pain (sudden stimulus–independent electric shock–like pain) was more prevalent in patients with thermal allodynia than in patients without allodynia No difference between CRPS type I and type II
Van Rooijen et al., 2012^53^	Cross sectional	IASP/Orlando (CRPS 1 with dystonia) and IASP/Budapest (research criteria) (CRPS 1 without dystonia)	N = 281 CRPS type I: 281 CRPS type II: 0	44.5 (SD 12.5)	59.4	NR	5.5 y (IQR 6.5–9.8)	Two subgroups: (1) With dystonia (2) Without dystonia	To test the genetic association with HLA-B62 and HLA-DQ8 in CRPS patients CRPS with and CRPS without dystonia may be genetically different but overlapping disease entities because only HLA-DQ8 is associated with both
Van Rooijen et al., 2013^51^	Cross sectional	IASP/Orlando	N = 48 CRPS type I: 48 CRPS type II: 0	46.7 (SD 12.1)	72.9	UE	9.3 y (IQR 3.5–14.7)	Two subgroups: (1) With dystonia (2) Without dystonia	To compare sensory function using QST between patients with dystonia, without dystonia, and healthy controls and to compare sensory functions with motor performance Both patients with and without dystonia had a loss of function to warmth detection, cold detection, and a gain of function to cold pain and pressure pain in the CRPS limb In addition, patients with dystonia experienced a loss of function for vibration detection and 2-point discrimination had a greater gain of function for pressure pain than patients without dystonia Patients with dystonia were slower at recognizing their affected limb than those without dystonia and had poorer finger tapping performance than those without dystonia. Pressure-pain thresholds correlated with performance on finger tapping in all 3 groups and with dystonia severity
Van Rooijen et al., 2013^52^	Cross sectional	IASP/Orlando	N = 48 CRPS type I: 48 CRPS type II: 0	46.4 (SD 12.1)	72.9	UE	10.0 (SD 7.4)	Two subgroups: (1) With dystonia (2) Without dystonia	To investigate sensory function using QST in unaffected limbs and cheeks of CRPS patients with and without dystonia and compare them with healthy controls Pressure hyperalgesia was the most prominent finding at all unaffected sites in both patients with and without dystonia. Patients with and without dystonia were similar apart from a lower vibration threshold in patients with dystonia
Vaneker et al., 2005^54^	Cross sectional	Veldman	N = 47 CRPS type I: 47 CRPS type II: 0	58 (SD 15)	70	UE	NR	Two subgroups: 1) Warm CRPS (mean ΔT = +0.65 in CRPS limb vs healthy limb) (2) Cold CRPS (mean ΔT = −0.78°C in CRPS limb vs healthy limb)	To identify altered patterns of sensory processing using QST 8 y after diagnosis, to document differences in sensory processing between warm and cold CRPS, and to determine relationships between changes in sensory processing and disease progression regarding pain Patients with cold CRPS had poorer clinical pain outcome, and more signs of central sensitization which increased with disease progression. Pressure-pain thresholds were lower in the CRPS limb in both groups and with disease progression
Varenna et al., 2016^55^	Cross sectional	IASP/Budapest (research criteria)	N = 194 CRPS type I: 194 CRPS type II: 0	57.1 (SD 12.9)	62.9	UE, LE	4 mo (range 2–6)	Two subgroups: (1) Warm CRPS (2) Cold CRPS Definition of warm vs cold not reported	To assess whether the effectiveness of bisphosphonates in CRPS patients is influenced by variables related to patient and/or disease characteristics Responsiveness to bisphosphonates was predicted by warm disease subtype, shorter CRPS duration, and fracture as initiating event
Zyluk et al., 2013^57^	Observational (registry)	IASP/Budapest	N = 220 CRPS type I: NR CRPS type II: NR	NR	NR	UE, LE	NR	Three subgroups: (1) Acute CRPS (2) Chronic CRPS (3) Chronic, refractory CRPS	Analysis of institutional CRPS register with particular attention to a chronic, refractory CRPS subtype that is extremely severe, has a disabling course, and is resistant to treatment All patients with chronic refractory CRPS were female, and all experienced neurological symptoms (hyperpathia, allodynia, tremor, muscle cramps, dystonia)

AR, adrenoreceptor; CRPS, complex regional pain syndrome; HLA, human leucocyte antigen; IASP, International Association for the Study of Pain; IL, interleukin; LE, lower extremity; MMP, metallproteinase; NR, not reported; PTSD, posttraumatic stress syndrome; QST, quantitative sensory testing; TNF, tumor necrosis factor; UE, upper extremity; ΔT, temperature difference.

### 3.3. Quality assessment

All included studies were nonrandomized, which allowed the use of the MINORS criteria for quality assessment. Scores averaged 69.3% (range, 37.5%–83.3%). Of the 25 studies included, 16 were classified as having low risk of bias,^1,4,9,11,13,18,21–23,25,26,30,51–54^ 8 studies with a moderate risk of bias,^8,14,15,19,20,38,39,55^ and 1 study with high risk of bias^57^ (Table 2).

**Table 2:** Individual risk of bias assessment using the methodological items for nonrandomized studies criteria.

Author, year (reference)	Clearly stated aim	Inclusion of consecutive patients	Prospective data collection	Endpoints appropriate to study aim	Unbiased assessment of study endpoint	Follow-up period appropriate to study aim	<5% loss to follow-up	Prospective calculation of study size	Adequate control group	Contemporary groups	Baseline equivalent groups	Adequate statistical analysis	Total score (%)	Risk of bias
Alexander et al., 2012^1^	2	1	2	2	1	2	0	2	2	2	2	2	83.3%	Low
Birklein et al., 2014^4^	2	1	2	2	2	2	0	0	2	2	2	2	79.2%	Low
Bruehl et al., 2002^8^	2	1	2	2	0	2	0	0	NA	NA	NA	NA	56.3%	Moderate
Bruehl et al., 2016^9^	2	1	2	2	2	2	1	0	NA	NA	NA	NA	75.0%	Low
De Boer et al., 2011^11^	2	2	2	2	0	2	2	0	N/A	N/A	N/A	NA	75.0%	Low
De Mos et al., 2009^13^	2	0	2	2	0	2	1	0	2	2	2	2	70.8%	Low
De Rooij et al., 2009^14^	2	0	2	2	0	2	0	2	2	1	1	2	66.7%	Moderate
De Rooij et al., 2010^15^	2	0	0	2	1	2	0	0	2	2	0	2	54.2%	Moderate
Dimova et al., 2020^18^	2	2	2	2	2	2	0	0	NA	NA	NA	NA	75.0%	Low
Dirckx et al., 2015^19^	2	0	0	2	2	2	0	0	2	2	0	2	58.3%	Moderate
Drummond et al., 2014^20^	2	0	2	2	1	2	0	0	2	2	0	2	62.5%	Moderate
Drummond et al., 2018^21^	2	0	2	2	1	2	2	1	NA	NA	NA	NA	75.0%	Low
Drummond et al., 2018^22^	2	0	2	2	2	2	0	0	2	2	2	2	75.0%	Low
Eberle et al., 2009^23^	2	2	2	2	2	2	0	0	2	2	2	2	83.3%	Low
Escolano-Lozano et al., 2021^25^	2	1	2	2	0	2	0	0	2	2	2	2	70.8%	Low
Gierthmuhlen et al., 2012^26^	2	1	2	2	1	2	0	0	2	2	1	2	70.8%	Low
Huge et al., 2008^30^	2	2	2	2	1	2	0	0	2	2	2	2	79.2%	Low
Libon et al., 2010^38^	2	2	2	2	0	2	0	0	NA	NA	NA	NA	62.5%	Moderate
Lunden et al., 2022^39^	2	0	0	2	2	2	0	0	NA	NA	NA	NA	50.0%	Moderate
Van Rooijen et al., 2012^53^	2	2	2	2	2	2	0	0	2	1	2	2	79.2%	Low
Van Roojen et al., 2013^51^	2	1	2	2	1	2	0	0	2	2	2	2	75.0%	Low
Van Roojen et al.,2013^52^	2	1	2	2	1	2	0	0	2	2	2	2	75.0%	Low
Vaneker et al., 2005^54^	2	2	2	2	0	2	1	0	2	2	2	2	79.2%	Low
Varenna et al., 2017^55^	2	2	0	2	2	2	0	0	NA	NA	NA	NA	62.5%	Moderate
Zyluk et al., 2013^57^	1	2	0	1	2	0	0	0	NA	NA	NA	NA	37.5%	High

### 3.4. Subtypes

Phenotyping in the included studies was based on the following variables: clinical presentation/sensory disturbances, dystonia, skin temperature (warm vs cold CRPS), disease duration, onset type (CRPS type I, CRPS type II, familial CRPS, spontaneous CRPS, traumatic CRPS), CRPS outcome, and neuropsychological test performance. Two studies investigated subtypes based on biomarkers of inflammation. Table 3 provides an overview of the clinical phenotyping variables and the studies that looked at the respective variables. It needs to be noted that some studies looked at more than one variable.

**Table 3:** Summary of the variables used to phenotype patients in the included studies.

Variables	Studies [references]
Clinical/sensory phenotyping	8,18,39
Dystonia	51–53
Skin temperature	9,19,22,23,54,55
Biomarkers of inflammation	1,25
Disease duration	4,11,21,22,30
Onset type	
CRPS type I and CRPS type II	21,22,26
Familial CRPS	14
Spontaneous CRPS	15
Traumatic CRPS	15
CRPS outcome	13,20,57
Neuropsychological test performance	38

CRPS, complex regional pain syndrome.

#### 3.4.1. Clinical/sensory phenotyping

Three studies examined clinical or sensory phenotyping variables and found support for CRPS subtypes based on these data, although the identified subtypes were not identical.^8,18,39^ In the first study,^8^ a cluster analysis based on the presence of signs/symptoms within the 4 CRPS diagnostic sign/symptom domains (pain/sensory abnormalities, vasomotor dysfunction, edema/sudomotor dysfunction and motor/trophic changes) led to 3 possible subtypes: (1) a predominately vasomotor subtype, (2) a predominately neuropathic pain/sensory abnormalities subtype, and (3) a subtype described as florid CRPS with the presence of broad CRPS features and the highest levels of motor/tropic signs together with osteopenia on bone scan in the affected limb. Pain duration did not differ between the 3 subtypes. Patients were mainly CRPS type I (67%). However, abnormalities on EMG/nerve conduction velocity testing were most common in the neuropathic/sensory abnormalities subtype, leading the authors to conclude that differences between the vasomotor subtype and the neuropathic/sensory abnormalities subtype may correspond to CRPS type I and CRPS type II, respectively (low risk of bias).

In another low risk of bias cluster analysis, this time based on objective CRPS signs only, Dimova et al.^18^ identified 3 primary CRPS subtypes: (1) a peripheral inflammation subtype that experienced edema, skin color changes, skin temperature changes, sweating, and tropic changes in the affected limb, (2) a central subtype reflecting CNS pathophysiology (minor injury, motor disturbances, allodynia, glove/stocking like sensory deficits) who also experienced cold hyperalgesia in the affected limb, and (3) a mixed subtype (combination of both subtypes above). The 3 subtypes did not differ in relation to age, CRPS duration, sex, or CRPS type I vs CRPS type II. Fewer patients with a cold limb were in the peripheral subtype (10%) than the mixed subtype (25.9%), and the central subtype had more cases of CRPS following minor injury than the peripheral and mixed groups (low risk of bias).

In a third clinical/sensory phenotyping study, Lunden et al.^39^ identified 3 CRPS subtypes based on the pattern of quantitative sensory testing (QST) results: (1) a subtype with temperature allodynia (to warm or cold), (2) a subtype with elevated warmth and cold detection thresholds (compatible with small fiber degeneration), and (3) a subtype with both elevated thermal detection thresholds and temperature allodynia. Paroxysmal pain (sudden stimulus–independent, electric shock–like pain) and allodynia to touch were more prevalent in patients displaying the thermal allodynia subtype (in particular cold allodynia) compared with patients without thermal allodynia. The authors attributed this to hyperexcitable superficial skin nociceptors and spinal central sensitization in these patients. Pain intensity and distribution of CRPS type I and type II did not differ between the 3 subtypes and, thus, pain appeared to be unrelated to small nerve fiber degeneration. The authors did not assess for any relation to disease duration (moderate risk of bias).

#### 3.4.2. Phenotyping based on dystonia

Differences between CRPS patients with vs without dystonia have been investigated in 3 studies.^51–53^ Van Rooijen et al.^53^ assessed whether patients with and without dystonia (all CRPS type I) differ genetically on alleles of the human leukocyte antigen system (HLA). Human leukocyte antigen-B62 was associated with CRPS with dystonia, whereas HLA-DQ8 was associated with both subgroups (compared with healthy controls), suggesting that CRPS subtypes with and without dystonia may be genetically different but overlapping disease entities (low risk of bias).

Another study examined the relation between sensory function assessed using QST and motor performance in CRPS with and without dystonia, as well as in healthy controls.^51^ The CRPS without dystonia subtype showed a loss of function to warmth detection, cold detection, and a gain of function to cold pain and pressure pain in the CRPS limb. The CRPS with dystonia subtype experienced a similar loss and gain of function but in addition experienced a loss of function for vibration detection and two-point discrimination and had a greater gain of function for pressure pain than the CRPS without dystonia subtype. Patients with the CRPS with dystonia subtype were also slower at recognizing the affected limb than those with the CRPS without dystonia and performed more poorly on finger tapping than those with the CRPS without dystonia subtype. Pressure-pain thresholds inversely correlated with performance on finger tapping in all 3 groups and with dystonia severity, suggesting that muscle hyperalgesia in the CRPS limb may contribute to motor impairments (low risk of bias).

To see whether sensory dysfunction is widespread throughout the body in CRPS patients and whether it relates to the presence of dystonia, the third study investigated sensory function using QST in unaffected body parts of CRPS patients.^52^ Widespread muscle hyperalgesia, in particular to pressure, was found in unaffected body parts. No differences were found between dystonia-related CRPS subtypes except for lower vibration thresholds relative to the unaffected leg of CRPS patients with dystonia (low risk of bias).

#### 3.4.3. Phenotyping based on skin temperature

Six studies investigated subtypes based on whether the skin temperature of the CRPS limb could be classified as warm or cold relative to the unaffected limb.^9,19,22,23,54,55^ In some studies, support for warm and cold CRPS subtypes was found.^9,19,23,54,55^ Definitions of what constitutes a warm and cold CRPS limb differed slightly between the studies, ranging from a difference of 0.60°C to 1.0°C between the CRPS limb and the contralateral limb (Table 1). One of the studies matched a group of patients with warm CRPS with a group of patients with cold CRPS in terms of age, sex, affected limb, CRPS duration, and type of CRPS (CRPS type I and CRPS type II).^23^ This study found that patients with the cold CRPS subtype more often reported serious life events, other chronic pain disorders, CRPS-related dystonia, cold-induced pain, and sensory loss on QST assessment in the CRPS limb, whereas patients with the warm CRPS subtype predominantly experienced mechanical hyperalgesia in the CRPS limb^23^ (low risk of bias).

Vaneker et al.^54^ used QST to explore differences in sensory processing between warm and cold CRPS subtypes 8 years after diagnosis. All patients had CRPS type I. Both the warm and cold subtypes exhibited pressure hyperalgesia in the affected limb and a worsening of this with disease progression. However, 8 years after diagnosis, patients initially diagnosed with the cold CRPS subtype had poorer clinical outcomes and showed persistent signs of central sensitization. Patients with cold CRPS also experienced more pain from electrical stimulation than those with the warm CRPS subtype (moderate risk of bias).

Three studies looked at the relation of skin temperature in the CRPS limb with inflammation.^19,22,55^ Dirckx et al.^19^ conducted a retrospective analysis of CRPS patients to assess signs of inflammation in patients with warm, cold, and intermediate temperature subtypes. Proinflammatory cytokine levels (tumor necrosis factor-alpha [TNF-alpha] and interleukin-6) were determined in fluid from artificially induced suction blisters made on the CRPS limb and the contralateral limb. The 3 subtypes did not differ in levels of these proinflammatory cytokines in the CRPS limb vs the contralateral limb. Nonetheless, compared with the warm CRPS subtype, the cold CRPS subtype consisted of younger patients with longer CRPS duration. The authors did not report whether patients were CRPS type I and/or CRPS type II (moderate risk of bias). In another study, a cluster analysis based on signs and symptoms of inflammation in predominantly CRPS type I patients (85%) found evidence for a warm CRPS subtype characterized by a warm, red, edematous, sweaty extremity, and a statistically distinct cold CRPS subtype marked by a cold, blue, and less edematous limb. Pain duration was again longer in patients with the cold CRPS subtype. However, 16% of patients in the cold CRPS group had a pain duration of <6 months. Patients initially displaying the warm CRPS subtype scored higher than the cold CRPS subtype on a clinically derived inflammation score, although inflammation decreased over the 3-month follow-up. This pattern was not observed in cold CRPS.^9^ The 2 subtypes did not differ regarding pain intensity (low risk of bias).

In another study addressing warm vs cold CRPS subtypes, Varenna et al.^55^ assessed whether the effectiveness of bisphosphonates in CRPS type I patients was influenced by several clinical and demographic factors, including warm vs cold subtypes. Responsiveness to bisphosphonates was predicted by displaying the warm CRPS subtype, as well as shorter CRPS duration and fracture as an initiating event (moderate risk of bias).

Drummond et al.^22^ evaluated whether upregulated cutaneous expression of α1-adrenoceptors (α1-AR) is a source of pain in CRPS patients by comparing patients and healthy controls. They also looked at CRPS subtypes based on limb temperature (warm, cold, indeterminate) among other subgroup analyses (CRPS duration, CRPS type I vs type II). α1-AR immunoreactivity was greater in nerve bundles of the reticular dermis (but not in the epidermis and blood vessels) of CRPS patients than healthy controls both in the CRPS limb and the contralateral limb. α1-AR immunoreactivity was also greater in the affected than in the contralateral limb. Greater α1-AR immunoreactivity of nerve bundles may be associated with pain in CRPS because α1-AR immunoreactivity was greater in dermal nerve bundles in the CRPS limb of a possible CRPS subtype that experienced evoked pain and pinprick hyperalgesia to injection of the α1-AR agonist phenylephrine. However, α1-AR immunoreactivity was similar in patients with the cold vs warm subtypes (and the undetermined thermal subtype) for all regions of interest (nerve bundles, epidermis, and blood vessels) (low risk of bias).

#### 3.4.4. Phenotyping based on markers related to inflammation

Two studies investigated subtypes based on markers related to inflammation.^1,25^ Alexander et al.^1^ evaluated plasma levels of cytokines, chemokines, and their soluble receptors in CRPS patients compared with gender-matched and age-matched healthy controls. Using cluster analysis, 2 distinct clusters were identified in CRPS patients: a subtype with levels similar to healthy controls (noninflammatory subtype) and a subtype with elevated levels of most plasma cytokines and soluble receptors (inflammatory subtype). There was no difference in CRPS duration between the 2 groups. However, in the inflammatory subtype, increased plasma levels of TNF-alpha correlated positively with disease duration. No difference in inflammatory markers between CRPS type I and CRPS type II subtypes was observed. Additional cluster analyses confirmed the positive correlations between TNF-alpha levels and both CRPS duration and severity in those patients with elevated levels of TNF-alpha who did not have an increase in its soluble receptor sTNF-RII. For patients with increased levels of the interleukin IL-1beta (and the interleukin soluble receptor sIL-1RI) without an increase in the interleukin soluble receptor sIL-1RII and the interleukin 1 receptor antagonist IL-1Ra, IL-1beta levels similarly correlated positively with both CRPS duration and severity (low risk of bias).

Escolano-Lozano et al.^25^ compared matrix metalloproteinases (MMP)-2 and MMP-9 concentrations in skin and serum of patients with CRPS, patients with other pain conditions, and healthy controls and related this to clinical data and QST results. The authors did not report the proportion of CRPS type I vs CRPS type II patients in the sample. These 2 enzymes play an important role in inflammation. Matrix metalloproteinases-2 was increased bilaterally in the skin of CRPS patients and MMP-9 in the ipsilateral CRPS skin relative to non-CRPS controls. Findings suggested that MMP-2 and MMP-9 are differently expressed depending on the clinical phenotype of CRPS; low ipsilateral MMP-2 was associated with trophic changes, and contralateral MMP-2 was associated inversely with CRPS severity. Higher ipsilateral and contralateral MMP-9 was associated with higher CRPS severity (low risk of bias). These patterns of MMP-2 and MMP-9 may support distinct inflammatory subtypes of CRPS.

#### 3.4.5. Phenotyping based on disease duration

Five studies explored CRPS subtypes related to disease duration.^4,11,21,22,30^ As mentioned earlier in the section on skin temperature phenotyping, Drummond et al.^22^ also evaluated upregulated cutaneous expression of α1-AR in relation to CRPS duration in a study with a low risk of bias. Three rationally derived duration categories were predetermined: (1) acute CRPS, <12 months; (2) intermediate CRPS, 11–36 months; and (3) chronic CRPS, >36 months. α1-AR immunoreactivity was greater in nerve bundles within the CRPS limb of patients with the acute CRPS subtype compared with both the intermediate and chronic CRPS subtypes but was similar across duration subtypes in the contralateral limb. Furthermore, α1-AR immunoreactivity was greater on dermal nerves in the CRPS limb than that in the contralateral limb irrespective of CRPS duration subtype. However, α1-AR immunoreactivity in the epidermis was greater bilaterally in patients with the acute or intermediate CRPS subtypes compared with the chronic subtype for patients with CRPS type II, whereas the opposite was seen in CRPS type I. Furthermore, α1-AR immunoreactivity on blood vessels was greater in the acute CRPS subtype than either the intermediate or chronic CRPS subtype.

Another study by Drummond et al.^21^ assessed hemisensory disturbances in relation to the same CRPS duration subtypes above (acute CRPS, <12 months; intermediate CRPS, 11–36 months; chronic CRPS, >36 months). In both, CRPS type I and CRPS type II, the distribution and intensity of mechanical hyperalgesia was similar in patients with acute, intermediate, and chronic CRPS, but patients with the chronic CRPS subtype experienced increased cold pain ratings and increased sharpness ratings to repeated pinprick in the ipsilateral forehead compared with the contralateral forehead. This was not seen in the acute or intermediate CRPS subtypes. Patients with chronic CRPS subtype was also more sensitive to thermal stimuli on both sides of their body than those with the acute or intermediate CRPS subtypes and was more likely to experience pain in an additional limb. These findings together suggest that heightened excitability of nociceptive pathways spreads centrally with increasing CRPS duration, for instance, to sensory convergence points in the brain stem or higher brain centers (low risk of bias).

De Boer et al.^11^ studied CRPS duration subtypes based on slightly different disease durations than Drummond et al.^22^ (<2 months, 2–6 months, 6–12 months, >12 months) and looked at the presenting signs and symptoms for each subtype in patients with CRPS type I. Sensory signs (allodynia, hyperalgesia) and motor signs (except limited range of motion) occurred more frequently in patients with a longer disease duration subtype (>6 months) than those with a short disease duration subtype (<2 months), with similar differences regarding changes in nail growth (but not skin or hair growth). Vasomotor and sudomotor signs were less frequent in patients with longer disease duration (>6 months) (low risk of bias).

Birklein et al.^4^ separated CRPS patients (type I and II) into 2 duration subtypes based on a 3-month CRPS duration cutoff (acute CRPS, <3 months, and chronic CRPS, >3 months) when looking at keratinocyte and mast cell proliferation in CRPS skin biopsies and identifying the cellular source of upregulated TNF-alpha, IL-6, and tryptase. Epidermal thickness and keratinocyte expression was increased in CRPS affected skin compared with the contralateral skin of patients with acute CRPS and decreased in affected skin of patients with chronic CRPS. Increases in TNF-alpha and IL-6 were more frequent in affected skin from acute CRPS patients than in those with chronic CRPS. Furthermore, tryptase-positive dermal mast cell numbers were increased in affected skin of acute CRPS patients but similar to the contralateral limb in patients with chronic CRPS. Temperature asymmetry (warm CRPS limb) correlated positively with the number of tryptase-labelled mast cells (low risk of bias).

Huge et al.^30^ defined CRPS duration subtypes based on the sequential stages proposed by Bonica^5^ with a time since inciting injury of 12 months or below defined as an acute CRPS subtype and a time since injury above 12 months as a chronic CRPS subtype. Thermal QST was investigated in the CRPS limb and the contralateral limb compared with a group of age-matched and gender-matched healthy controls. Patients with the acute CRPS subtype experienced warm and cold hyperalgesia, as well as warm and cold hypoesthesia, in the CRPS limb relative to the contralateral limb. Thermal hyperalgesia was not as severe as in the chronic CRPS subtype, and warm and cold hypoesthesia were more severe in the chronic CRPS subtype compared with acute CRPS. Only patients with the acute CRPS subtype experienced paradoxical heat sensations. All QST changes were also somewhat present in the contralateral limb relative to healthy controls. The difference in skin temperature between the acute and chronic CRPS subtypes was significant with a warmer CRPS limb in the acute subtype and a colder CRPS limb in the chronic subtype compared with the contralateral limb (low risk of bias).

#### 3.4.6. Phenotyping based on onset type

Five studies considered whether the type of initiating event or injury including major peripheral nerve lesion (CRPS type I vs CRPS type II) are relevant for subtyping patients.^14,15,21,22,26^

In the previously mentioned study by Drummond et al.^22^ who investigated the involvement of α1-AR in CRPS, subtyping by CRPS type I vs CRPS type II was also assessed. On average, α1-AR immunoreactivity in CRPS-affected skin was greater in nerve bundles of patients with CRPS type II than in those of patients with CRPS type I, particularly within the distribution of the injured nerve. However, no difference was seen for the contralateral limb. Furthermore, α1-AR immunoreactivity was greater on dermal nerves in the CRPS limb than the contralateral limb irrespective of CRPS type I or CRPS type II. Both patients with CRPS type I and CRPS type II were among the subgroup of patients who experienced increased pain and hyperalgesia to phenylephrine injection. As mentioned above, a difference in α1-AR immunoreactivity over time was noted across the CRPS type I and CRPS type II subtypes, with α1-AR immunoreactivity in the epidermis being greater bilaterally in CRPS type II patients with the acute or intermediate subtype compared with patients with chronic CRPS type II, whereas the opposite pattern over time was seen in CRPS type I. α1-AR immunoreactivity on blood vessels was greater in both limbs of patients with CRPS type II compared with patients with CRPS type I (low risk of bias).

In the study on hemisensory disturbances described previously, Drummond et al.^21^ also included CRPS type I and CRPS type II as formal subtypes. Patients with the CRPS type I subtype were more often female and more often had pain in more than one limb than those with the CRPS type II subtype. Sensory deficits and allodynia were more common in the CRPS-affected limb in patients with CRPS type II than in those with CRPS type I, but CRPS type I patients with the chronic CRPS subtype more often had pressure-pain sensitivity and allodynia in the uninjured limb on the symptomatic side of the body than in patients with chronic CRPS type II. Hyperalgesia to repeated pinprick was greater in the forehead ipsilateral to the CRPS limb than contralaterally in both chronic CRPS type I and chronic CRPS type II but not in intermediate and acute CRPS. Thermal thresholds and thermal ratings were similar in CRPS type I and CRPS type II (low risk of bias).

Gierthmühlen et al.^26^ also investigated differences in somatosensory signs between CRPS type I and CRPS type II subtypes using a full QST battery in the affected limb. Patients with CRPS type I and type II had almost identical sensory profiles in terms of hyperalgesia, allodynia, and thermal detection thresholds, although a loss of mechanical detection occurred more frequently in patients with the CRPS type II subtype. Correlations between QST findings and symptom duration were only modest. Hyperalgesia/allodynia without loss of detection (only gain) was more common in the CRPS type I subtype than in the CRPS type II (low risk of bias).

De Rooij et al.^14^ studied a possible familial CRPS subtype by evaluating families with a history of CRPS and comparing clinical characteristics of these patients with those of nonfamilial CRPS. Overall, patients with the familial CRPS subtype had younger age at onset, CRPS that more often affected multiple extremities, and more frequent dystonia. The study did not report whether patients had CRPS type I or CRPS type II (moderate risk of bias).

In another study by de Rooji et al.,^15^ a cross-sectional exploration to compare phenotypic characteristics of patients with spontaneous CRPS vs trauma-induced CRPS was performed. Findings suggested that patients with the spontaneous CRPS subtype were younger at CRPS onset and had a longer disease duration. Clinical presentation and sex distribution were similar in both subtypes. Again, the authors did not address whether patients had CRPS type I or CRPS type II (moderate risk of bias).

#### 3.4.7. Phenotyping based on clinical outcome

In 3 studies, possible CRPS subtypes were based on the long-term clinical outcomes of CRPS.^13,20,57^ de Mos et al.^13^ employed cluster analysis to derive 3 subtypes based on CRPS outcome (best, moderate, and poor) as reflected in the number of signs and symptoms that were present 2 years or more after the onset in a group of mainly CRPS type I patients (97%). Patients with the poor outcome subtype more often had upper extremity CRPS and a cold CRPS phenotype and less often reported fractures as the initiating event. Patients with the poor outcome subtype also reported ongoing disease progression and adjustments in employment (ie, stopped working or working with adaptations). All patients with the poor outcome subtype still fulfilled the IASP CRPS criteria at the time of follow-up assessment. No difference in CRPS duration was observed between the outcome groups (low risk of bias).

Drummond and Finch^20^ used a cross-sectional design to determine whether clinical characteristics differed between 2 CRPS subtypes: (1) patients who experience a pain increase in the CRPS limb to forehead cooling and (2) patients who do not experience a pain increase to forehead cooling. Patients who experienced a pain increase to forehead cooling also experienced greater pain in the CRPS limb before forehead cooling and were in general more sensitive to stimuli on the forehead, in particular on the side ipsilateral to the CRPS limb, and were more sensitive to pressure pain in the limbs. Together, this led the authors to conclude that the pain increase group was experiencing a disturbance in central somatosensory processing and pain modulation. Pain duration was greater in patients with this central somatosensory disturbance subtype. The subtypes did not differ in terms of proportion of CRPS type I vs CRPS type II (moderate risk of bias).

Finally, through an observational review of an institutional CRPS registry, Zyluk and Puchalski^57^ identified acute, chronic (3–6 months after onset), and refractory CRPS subtypes. Specifically, findings highlighted the importance of recognizing the chronic refractory subtype because of its extremely severe disabling course and resistance to treatment. The authors noted that their refractory subtype group consisted exclusively of women aged 18 to 40 years, and neurological symptoms were always present (hyperpathia, allodynia, tremor, muscle cramps, dystonia). However, this study had a high risk of bias.

#### 3.4.8. Phenotyping based on neuropsychology

One study investigated and found support for neuropsychological subtypes in CRPS. Libon et al.^38^ conducted a battery of tests that assess executive function, naming/lexical retrieval, and declarative memory in CRPS patients. Based on a two-step cluster analysis, the following 3 subtypes were identified: (1) a cognitively normal subtype with scores in the average range on all tests, (2) a dysexecutive subtype with mild impairment or low average performance on working memory/verbal fluency tests, and (3) a global cognitive dysfunction subtype with scores in the low average/borderline range on all tests but with particularly low scores on naming/declarative memory tasks. The latter 2 groups were equally impaired on executive function tests. The global dysfunction CRPS subtype presented with fewer years of education compared with the other groups and with a higher score on the Beck Depression Inventory-II than the cognitively normal subtype. However, these differences in education and depression levels did not statistically account for the differences in neuropsychological variables. Overall, the 3 cognitive subtypes did not differ in terms of pain levels, CRPS duration, medication use, or the number of CRPS-affected limbs. The authors did not report on the distribution of CRPS type I and CRPS type II (moderate risk of bias).

## 4. Discussion

This review systematically identified and summarized studies investigating possible subtypes of CRPS. The findings from the included studies provide support for the following subtypes: CRPS type I, CRPS type II, acute CRPS, chronic CRPS, centralized CRPS, cold CRPS, warm CRPS, inflammatory CRPS, dystonic CRPS, nondystonic CRPS, familial CRPS, and nonfamilial CRPS. It is unclear whether these are distinct or overlapping subtypes. There was also limited evidence that there may be CRPS subtypes with distinctive impairments in cognitive function.

Surprisingly, only a few of the studies aimed to formally assess differences between CRPS type I and CRPS type II patients, although many of the studies have mentioned the contribution of CRPS type I and CRPS type II to their findings. Absence of formal comparisons may in part be due to the much smaller samples of CRPS type II patients in most studies. Some studies did not report or distinguish between whether patients had CRPS type I or CRPS type II. There were indications from the studies that CRPS type I and CRPS type II may be relevant subtypes. For instance, different mechanisms may contribute to the upregulation of adrenoceptors in CRPS type I and CRPS type II patients as α1-AR immunoreactivity in the skin was differentially expressed in CRPS type I and CRPS type II patients and was associated with CRPS duration in different directions in the 2 groups.^22^ A difference in clinical presentation with vasomotor signs and symptoms being more common in CRPS type I, and neuropathic/sensory abnormalities more common in CRPS type II^8^ also point to possible differences in some of the mechanistic underpinnings. Consistent with this, sensory loss to mechanical stimuli was more frequent in patients with CRPS-type II than CRPS-type I.^22,26^ However, there was disagreement as to whether allodynia is more frequent in CRPS type II than in CRPS type I^22,26^ or of similar frequency.^26^ It is possible that the greater sensory loss in CRPS type II arises as a result of the peripheral nerve lesion leading to the type II diagnosis.^26^ However, peripheral nerve damage, albeit of small nerve fibers, has also been suggested to occur in CRPS type I.^42,44^ Nonetheless, damage to small fibers would be expected to lead to sensory loss to thermal QST tests (small fibers) rather than mechanical stimuli (large fibers), which did not differ between CRPS type I and type II.^26^ The relevance of the CRPS type I and CRPS type II subtype nomenclature has been debated ever since the terms replaced the former diagnoses of reflex sympathetic dystrophy (CRPS type I) and causalgia (CRPS type II). During the development of the Budapest criteria, there were considerations to remove this division because there was broad agreement that the clinical presentation and therapeutic response was not significantly different.^29^ Primarily for historical reasons, the terms were retained.^29^ Findings in this review provide a rationale for further research into differences between CRPS type I and CRPS type II. Such studies need to carefully differentiate patients with CRPS type II from those with posttraumatic neuralgia, which may display similar sensory–motor changes albeit confined to the lesioned nerve territory. For patients with CRPS type II, sensory changes must go beyond the territory of the lesioned nerve.

Regardless of CRPS-type I vs CRPS-type II subtype status, sensitivity outside the CRPS limb seemed to intensify in patients with chronic/persistent CRPS, which points to changes in the nociceptive system centrally with increasing disease duration irrespective of CRPS type I or II.^21,26^ Some work suggest that this centralization with chronicity may be more common in CRPS-type I because patients with chronic CRPS type I had a greater spread of pain and hyperalgesia outside the CRPS limb ipsilaterally compared with chronic CRPS type II.^21^ Also consistent with the occurrence of central changes with chronicity, de Boer et al.^11^ found allodynia, hyperalgesia, and motor signs to be more frequent in the CRPS limb of patients with a longer CRPS duration. The finding of a central disturbance in somatosensory processing (perhaps a switch from inhibition of nociception to facilitation) in a subgroup of patients with a longer pain duration irrespective of CRPS type I or II provide further support for central changes in persistent CRPS.^20^ The presence of pain and increased sensitivity outside the CRPS-affected limb in chronic/persistent CRPS, as noted above,^21,26^ may reflect the nociplastic process of central sensitization.^49,56^ At which time point during the disease course these central aspects come into play is unclear and needs to be investigated further. In the included studies, disease durations that defined chronic CRPS differed widely from >3 months to 66 months, and some of the studies referred to pain duration rather than CRPS duration.

Other studies outside of this review have also found evidence for disturbances in central endogenous pain inhibition in CRPS,^34,47^ but some have found that these processes remain intact (ie, intact conditioned pain modulation). In some studies, these disturbances correlated positively with pain duration,^34^ and, in other studies, they did not.^47^ Thus, there is a need for more studies into possible alterations in central pain inhibitory systems. In defining subtypes, it may be more valuable to focus on a subtype reflecting presumed centralized mechanisms based on clinical features rather than a subtype defined by an arbitrary pain duration cutoff because a centralized pathophysiology may exist in a subgroup of CRPS patients irrespective of chronicity. In the study by Dimova et al.,^18^ a statistically distinct centralized pathophysiology subtype appeared to be unrelated to CRPS duration. The presence of thermal allodynia in a subgroup of patients in association with touch allodynia and paroxysmal pain also support the existence of a centralized CRPS subtype,^39^ although this study did not examine the contribution of CRPS duration to these findings. It is possible that CNS pathophysiology is a potential risk factor for developing CRPS in some patients. This would be consistent with prospective studies suggesting that elevated central sensitization as indexed by more severe pain intensity^41^ and elevated temporal summation to pain during QST^7^ predicts development of CRPS following tissue trauma. The higher prevalence of CRPS following minor injury in the centralized group compared with the peripheral inflammation group or mixed group in the study by Dimova et al.^18^ also provides some support for this. However, one cannot exclude the possibility that differences in samples and how duration was estimated (pain or disease) in the studies may explain why a link with CRPS duration was found in some but not other studies. Differences in findings between the studies may also arise because of differences in whether the statistical analyses or between-group analyses performed were correlational.

The presence of motor disturbances together with allodynia in the centralized CRPS group^18^ is in line with the link between greater muscle hyperalgesia and motor impairments (dystonia severity and poorer finger tapping performance) in one of the other studies,^51^ and together,they suggest that circuitries mediating nociception may play a role in impaired motor control/dystonia in CRPS, perhaps through both peripheral and central sensitization. These findings are also in line with findings that motor cortex stimulation may alleviate chronic pain in CRPS and other pain conditions.^43^ However, this will need to be investigated further because patients with and without dystonia did not seem to differ in terms of widespread muscle hyperalgesia.^52^

Limited evidence from one study was also found for a cognitive impairment CRPS subtype, which appeared to be unrelated to pain intensity or CRPS duration and widespread limb involvement.^38^ Other studies have also reported impairments in cognitive processing related to tactile and emotional decision making in small samples of CRPS patients^2,36^ who were unrelated to pain duration or psychological distress,^2^ but these latter studies did not address the presence of a possible CRPS subtype reflecting these impairments.^2,36^ Thus, more research is needed into a possible cognitive impairment subtype in CRPS. Whether and how impairments in cognition may relate to the other signs of CNS pathophysiology or CRPS outcomes remain to be investigated, but it is well documented that patients with CRPS may experience cortical changes in brain areas associated with somatosensory and motor processing,^16,17,37,50^ which could potentially interfere with cognition.^32^

The fact that some mechanisms contribute to severe disease in a subgroup of patients independent of disease duration was supported by a number of the included studies.^8,13,57^ Factors that seemed to distinguish people with poor outcome from the remaining patients were a cold upper extremity CRPS limb with an initiating event other than a fracture rather than CRPS duration.^13,54^ Neurological symptoms, including hyperpathia, allodynia, dystonia, tremor, and muscle cramps, were furthermore argued to always be present in patients with severe CRPS refractory to treatment.^57^ However, the latter study had a high risk of bias, and thus, this will need to be replicated in a larger controlled study. Whether such changes reflect a distinct CRPS subtype remains to be determined.

Besides a poorer clinical prognosis, the included studies together suggest that the cold CRPS subtype may differ from patients with the warm CRPS subtype by more often having serious life events, other chronic pain disorders, CRPS-related dystonia, sensory loss to QST in the CRPS limb, and more pain from electrical stimulation, whereas warm CRPS predominately experienced mechanical hyperalgesia in the CRPS limb.^23,54^ It has been speculated whether there is an overlap between cold CRPS and persistent CRPS as a number of studies found patients with a cold limb to have a longer pain duration than those with warm CRPS^9,19,30^ and signs of central sensitization,^54^ although some patients were found to have a cold limb in the early CRPS stage.^9^ Similarly, warm CRPS has been postulated to overlap with acute CRPS.^27^ The finding in one of the included studies that mast cell numbers were increased in affected skin from patients with acute CRPS and that this correlated with a warmer CRPS limb provide some support for this.^4^ Mast cell degranulation releases a range of inflammatory mediators such as tryptase, proteases, histamine, and cytokines.^24^ The link between acute and warm CRPS may be explained by histamine-induced vasodilation. Nonetheless, there does not seem to be enough evidence yet to fully confirm the overlaps between persistent/cold and early/warm CRPS, but the evidence points to cold and warm CRPS and acute and persistent CRPS as relevant subtypes.

Some have speculated whether cold and warm CRPS differ in terms of inflammation, but the evidence regarding this is unclear. A cluster analysis of signs and symptoms of inflammation provided support for a greater involvement of inflammation in warm CRPS,^9^ and greater thermal hyperalgesia to heat in warm/acute than in cold/persistent CRPS are also consistent with peripheral sensitization of heat-sensitive C-fibers during inflammation in warm CRPS.^30^ Nonetheless, no differences in the proinflammatory cytokines TNF-alpha and interleukin-6 in the CRPS limb was found between warm and cold CRPS.^19^ Conversely, bisphosphonates were found to have the best effect in early warm CRPS, and as bisphosphonates have been shown to have anti-inflammatory effects, for instance, in rheumatoid arthritis,^32^ this may provide support for inflammation in early warm CRPS. It is possible that different definitions used for warm vs cold CRPS may explain differences in findings because no consensus has been reached on how to define warm and cold CRPS. Such definitional issues warrant systematic examination in future research.

Although it is unclear whether inflammation contributes to differences between the warm and cold CRPS subtypes, there is considerable evidence to suggest the involvement of inflammation in a subgroup of patients with CRPS.^18^ A peripheral inflammation subtype emerged based on signs of CRPS.^18^ Interestingly, a minority of these inflammation subtype patients experienced cold CRPS, which provide some support for inflammation being primarily present in the early warm CRPS. Furthermore, in the study of plasma cytokines, chemokines, and their soluble receptors by Alexander et al.,^1^ 2 clusters of patients emerged: one with levels similar to healthy controls and another with elevated levels of almost all analytes. Tumor necrosis factor-alpha appeared to be important for this clustering and may be relevant to CRPS subtyping efforts. There was no difference in duration and severity between the 2 clusters, arguing against inflammation as something primarily present in acute CRPS. However, within the elevated TNF-alpha group, plasma levels of TNF-alpha seemed to increase with disease severity/duration. Unfortunately, Alexander et al.^1^ did not look at the contribution of warm vs cold CRPS subtype characteristics as to how they may have related to TNF-alpha patterns. Consistent with the finding that TNF-alpha levels may change in some patients with disease duration, one of the other studies found increased TNF-alpha and IL-6 immunostaining on keratinocytes in affected skin (compared with the contralateral limb) to be more frequent in patients with acute than chronic CRPS.^4^ The opposing directional relationship between TNF-alpha and disease duration in the 2 studies may arise due to methodological differences (plasma vs skin biopsy) or the dissimilar subgroup focus (normal inflammatory profile/inflammatory profile vs acute/chronic). Studies that have investigated both local (skin biopsies or suction blister fluid) and systemic (plasma, serum) levels of cytokines, including TNF-alpha in CRPS, have found local but not systemic proinflammatory cytokine increases.^31,35^ Future studies should assess the relationship between local and systemic cytokine levels and their relation to disease duration and severity in CRPS. The finding that MMP-2 and MMP-9 enzymes known to be involved in inflammation are differentially expressed depending on the presence of trophic changes and disease severity in one of the included studies,^25^ provide further support for an inflammatory subtype that is more severe, but this was unrelated to CRPS duration.

Future studies should attempt to discern whether warm, acute, and inflammatory subtypes reflect the same subtype and if so, determine the optimal criteria for categorizing these patients. One may expect an objective mechanistic marker such as a biomarker of inflammation to be a better indicator than the more arbitrary marker of pain duration. Similarly, studies should look into whether there is an overlap between centralized and chronic cold CRPS and, if so, whether there is an objective mechanistic marker for this subtype.

Finally, evidence was found for a heritable component playing a role in a subgroup of patients with CRPS (dystonia and familial) and that this may contribute to a more severe and earlier onset CRPS (eg, dystonia, multiple affected extremities), but this will need to be investigated further.

This was the first review to systematically investigate the evidence for subtypes in CRPS. We were able to identify a small number of relevant studies. The studies approached the investigation of subtypes in CRPS in very different ways, and most had a low risk of bias but with small samples. Furthermore, the same diagnostic criteria were not used in all studies. Findings of de Boer et al.^11^ highlight that the diagnostic criteria used may substantially change the findings and conclusions drawn. Thus, substantially more research is needed using current IASP diagnostic criteria to draw any firm conclusions about subtypes of CRPS. We took a systematic approach to covering the literature regarding subtypes but cannot rule out that some studies may have been omitted because we were unable to discern from the title or abstract that subgroups were analyzed. Furthermore, we had to rely solely on the information reported in the studies reviewed, which sometimes lacked data regarding patient characteristics.

Notably, only one of the studies was an interventional study.^22^ Whether phenotyping CRPS patients into the identified subtypes has clinical relevance for predicting treatment outcome is yet to be determined. We recommend that future interventional studies examine the subtypes identified in this review because they are likely to impact on the responsiveness to treatments evaluated, and we recommend that future interventional studies seek to identify the phenotypic characteristics of treatment responders regardless of whether these characteristics fit within these identified subtypes. This is also a recommendation put forth in the ACTTION guide to clinical trials of pain treatment by the International Association for the Study of Pain.^10^

It is important conceptually to keep in mind that the subtypes described in this review may not be completely distinct from one another, which may complicate phenotyping. It is possible that the subtypes of CRPS may overlap within individual patients or coexist on different continuums within a patient. Nonetheless, the hope is that establishing clinically relevant subtypes may support identification of more effective treatments for CRPS within a precision medicine context. In other words, conducting clinical trials focused on a priori subtypes believed to reflect mechanisms, likely to respond to the presumed intervention mechanisms, may lead to success in clinical trials even where trials in large heterogeneous CRPS samples have failed.

## Disclosures

The authors have no conflict of interest to declare.
